# Assessing the hidden dangers of volcanic CO_2_ exposure: a critical review of health impacts

**DOI:** 10.3389/fpubh.2024.1465837

**Published:** 2024-10-04

**Authors:** Luis D. Boada, Katherine Simbaña-Rivera, C. Rodríguez-Pérez, M. Fuentes-Ferrer, Luis Alberto Henríquez-Hernández, E. López-Villarrubia, E. E. Alvarez-León

**Affiliations:** ^1^Toxicology Unit, Research Institute of Biomedical and Health Sciences (IUIBS), University of Las Palmas de Gran Canaria (ULPGC), Las Palmas de Gran Canaria, Spain; ^2^Centro de Investigación para la Salud en América Latina (CISeAL), Facultad de Medicina, Pontificia Universidad Católica del Ecuador (PUCE), Quito, Ecuador; ^3^Canary Health Service, University Hospital Nuestra Señora de Candelaria and Primary Care Authority of Tenerife, Santa Cruz de Tenerife, Spain; ^4^General Public Health Directorate, Canarian Health Service, Las Palmas de Gran Canaria, Spain; ^5^Spanish Consortium for Research on Epidemiology and Public Health (CIBERESP), Instituto de Salud Carlos III, Madrid, Spain; ^6^Preventive Medicine Service, Complejo Hospitalario Universitario Insular Materno Infantil, Canary Health Service, Las Palmas de Gran Canaria, Spain; ^7^Instituto Ramón y Cajal de Investigación Sanitaria (IRYCIS), Madrid, Spain

**Keywords:** volcanic CO2 emissions, health risks, chronic exposure, public health, mitigation strategies

## Abstract

Volcanic eruptions pose significant health risks to inhabitants of affected regions, with volcanic gases, including carbon dioxide (CO_2_), being a notable concern. This review examines the implications of long-term exposure to volcanic CO_2_ emissions on public health, highlighting the shift in understanding from acute to chronic health effects. Recent studies have underscored the need to reevaluate the adverse health impacts of CO_2_ beyond acute toxicity symptoms. While previous guidelines deemed an indoor (residential) acceptable long-term exposure range (ALTER) of ≤3,000 parts per million (ppm) in residential housing areas, emerging evidence suggests that even concentrations within the range of 3,000 to 1,000 ppm may induce deleterious health effects. International agencies now advocate for lower safe indoor CO_2_ levels (600–1,000 ppm), necessitating a reassessment of public health strategies in volcanic areas. This review argues for increased awareness among local and public health authorities about the chronic toxicity of CO_2_ exposure and emphasizes the importance of safeguarding populations from the adverse health effects induced by CO_2_ exposure.

## 1 Introduction

Volcanism stands as one of the most potent geological phenomena, exemplifying the dynamic activity within the Earth’s interior and the shifts of its crust (1). During volcanic eruptions, vast quantities of pyroclastic material, ashes, and gases are expelled, often projected over considerable distances (2). Intriguingly, around 500 million people globally reside within potential exposure zones of volcanoes, which can also induce climatic changes with global ripple effects (3, 4).

Among the array of volcanic gases, carbon dioxide (CO_2_) is notably prevalent in volcanic and geothermal regions worldwide (5). It is emitted in substantial quantities by soils around active and post-eruptive volcanoes (6, 7). While CO2, a naturally occurring trace gas in the atmosphere, is involved in essential processes such as cellular respiration, organic matter fermentation, and the combustion of fossil fuels, its elevated concentrations can be hazardous (8). The current atmospheric concentration of CO_2_ is approximately 412 parts per million (ppm), or about 0.04% (9). Elevated levels of CO_2_ can lead to intoxication by impacting oxygen and serum bicarbonate levels (10), and it is classified as an asphyxiant by international standards (11).

The implications of CO_2_ on human health extend beyond asphyxiation (12); it poses significant risks to human health, manifesting both acute and chronic adverse effects (13). Considering that volcanic zones may continuously emit CO_2_ across vast areas over extended periods, it becomes imperative to assess the health risks for potentially exposed populations (14). This exposure is notably prevalent in occupational settings such as metallurgy, welding, and the production of carbonated beverages, where CO_2_ is commonly encountered (15, 16).

From a toxicological view, CO_2_ is absorbed passively through the lungs and is primarily excreted via the lungs after being transported in the blood as bicarbonate, a process facilitated by the enzyme carbonic anhydrase (17).

Cases of CO_2_ poisoning have been documented in medical literature since the 1950s (18), often resulting from accidental exposure to CO_2_ from sources such as dry ice, fermentation, or inadvertently opened liquid CO_2_ tanks (19). Additionally, cases of CO_2_ poisoning stemming from suicide attempts or even homicides have been reported (20). Notably, fatalities from asphyxiation due to the accumulation of CO_2_ from volcanic sources have also been recorded (3, 5, 21).

Populations residing in volcanic regions, whether active or dormant, face significant risks from diffuse CO_2_ emissions. Anomalous gas emissions are a common occurrence in areas with recent volcanic activity (7, 22, 23). Particularly vulnerable are areas where residential and tourist activities converge near emission sites, such as Colli Albani (Italy), and Stromboli and Vulcano Islands (Aeolian Archipelago, Italy) (23, 24), Rotorua city in New Zealand (25), La Palma Island in the Canary Islands (Spain) (26), and Sao Miguel Island in Azores Archipelago (Portugal) (27). Some of these areas feature sites of continuous high gas emission, with intermittent periods of increased release.

Considering these factors, the aim of this study is to assess the adverse effects experienced by populations inadvertently exposed to volcanic CO_2_. Given the scarcity of comprehensive data on health risks associated with volcanic CO_2_ exposure beyond its fatal outcomes, this critical review synthesizes both scientific and gray literature to assess the chronic and acute health risks to populations exposed to variable CO_2_ concentrations.

## 2 Toxic effects of CO_2_: current understanding

As previously discussed, CO_2_ is not inherently toxic, although it could also act as a toxicant. It constitutes a component of air (412 ppm), and it is generated and employed in many human processes.

The gas’s odorless and colorless nature renders detection without specific testing devices impossible, and its high density in comparison to oxygen and nitrogen results in high concentrations being condensed in the lower layers of both indoor and outdoor air, heightening its danger. CO_2_ may accumulate in lower spaces, leading to oxygen deficiency. Consequently, CO_2_ concentrations tend to be higher in environments such as mines, sugar refineries, distilleries, grain silos, and drains (28).

Although CO_2_ emissions from volcanic sites can indeed be fatal, such incidents are rare. However, it is crucial to remember that CO_2_ was implicated in the deaths of approximately 1,700 people in Cameroon, West Africa, in 1986 (as well as a similar event at Lake Monoun, also in Cameroon, in 1984) following a massive release of gas from these volcanic crater lakes (29, 30). Similarly, on February 20, 1979, in Dieng Plateau (Indonesia), 149 people perished and 1,000 others were injured after being enveloped in a gas cloud produced by a phreatic eruption (24, 31).

In addition to these major incidents, recent fatal accidents in Western countries have also been attributed to exposure to high concentrations of volcanic-origin CO_2_. Notably, during a period of significant degassing at La Fossa crater in the 1980s, two children and numerous small wild animals lost their lives on Vulcano Island, Italy. During this time, emissions reached up to 1,350 tons per day, with outdoor CO_2_ concentrations recorded as high as 9.8% (98,000 ppm) (32). Moreover, D’Alessandro and Kyriakopoulos (2013) reported three additional fatalities in the 1990s linked to CO_2_ accumulation in the Pausanias thermal baths on the northern coast of the Methana Peninsula (Greece) (14). In 1992, two fatalities were reported in the Azores archipelago, Portugal, inside a lava cave where diffusely released CO_2_ reached indoor concentrations of 10% (100,000 ppm) (21). Furthermore, in 1998, other fatality was reported in Mammoth Mountain (California, United States) when a cross-country skier fell into a snow well where CO_2_ concentration soared to 70% (33). Interestingly, in some of these cases, the recorded CO_2_ concentrations were not excessively high, suggesting that even initial exposure to low concentrations can be hazardous. This is attributed to the fact that exposure to gradually increasing CO_2_ concentrations, starting from atmospheric levels, can induce unconsciousness due to the narcotic properties of CO_2,_ often without the victims perceiving any imminent danger (34, 35).

Additionally, the significant tourist activity in volcanic regions and geothermal fields globally necessitates attention to the risks faced by tourists in degassing zones. This is particularly crucial in coastal areas where tourists might be sunbathing close to the ground, a position that increases the risk of inhaling concentrated CO_2_ in the absence of wind. Monitoring activities that could lead to exposure to high CO_2_ concentrations is therefore essential for the safety of visiting tourists. Notably, cases of intoxication among tourists have been documented in volcanic regions. Documented cases of CO_2_ intoxication among tourists in volcanic regions underscore this risk. For instance, in April 2015, a 9-year-old French child experienced severe intoxication from gases emitted by an undersea vent near the shoreline on Vulcano Island, Italy (36). In 2019, three fatalities were reported among tourists visiting the “Solfatara,” geothermal area near Naples, Italy (37). Moreover, tourists in Sao Miguel (Azores Islands, Portugal), Hawaii (Hawaiian Islands, United States), and Mammoth Mountain (California, United States) have also suffered adverse health effects due to CO_2_ exposure (38–41).

The intoxications mentioned above can be attributed to the fact that CO_2_ poisoning typically involves life-threatening hypoxia and hypercapnia, resulting in varying levels of consciousness impairment ranging from drowsiness and confusion to deep coma and respiratory acidosis. In cases of inadvertent exposure to increasing CO_2_ levels, severe hypercapnia can lead to cerebral edema and respiratory center paralysis. Podlewski et al. have stated that CO_2_ concentrations of up to 1% (10,000 ppm) may induce drowsiness in some individuals, while concentrations above 3% (30,000 ppm) can disrupt gas exchange at the pulmonary membrane, altering pH and causing hypercapnia associated with brain damage and loss of consciousness. Breathing air with CO_2_ concentrations exceeding 5% (50,000 ppm) can lead to breathlessness, anxiety, and stimulation of the respiratory center. At levels between 7 and 10%, individuals may experience dizziness, headaches, visual and auditory impairments, and may rapidly become unconscious. It has been documented that CO_2_ concentrations of 9% inhaled for more than 10 min, and higher concentrations inhaled for less than 10 min, are poorly tolerated or not tolerated at all due to symptoms including exhaustion, anxiety, dissociation, or acidosis (pH < 7.2), despite normal oxygenation (42). Concentrations exceeding 10% are known to cause hallucinations and significantly impaired consciousness, potentially resulting in coma and convulsions. Furthermore, exposure to concentrations above 20% (200,000 ppm) typically results in death within minutes, while levels exceeding 30% (300,000 ppm) lead to instantaneous death (43). Acute effects of CO_2_ intoxication are shown in Table 1.

**Table 1:** Short-term effects based on CO_2_ concentration levels.

CO_2_ concentration (ppm)	Short-term effects
1,000–10,000	Drowsiness, headache, fatigue, concentration difficulties
10,000–30,000	Dizziness, headache, visual and auditory impairments, rapid onset of unconsciousness
30,000–50,000	Severe hypercapnia, breathlessness, anxiety, stimulation of the respiratory center
50,000–98,000	Increased risk of unconsciousness, severe intoxication symptoms (e.g., exhaustion, anxiety, dissociation, acidosis with intact oxygenation)
>100,000	Hallucinations, impaired consciousness, coma, convulsions
>200,000	Death within minutes
>300,000	Instant death

ppm, parts per million.

International recommendations and occupational guidelines regarding indoor CO_2_ levels suggest a time-weighted average (TWA) of 5,000 ppm (0.5%; for an 8-h weighted average) or a short-term TWA (15 min) of 30,000 ppm (44, 45). However, considering that elevated CO_2_ levels may disrupt placental development, it is essential to provide specific protection for pregnant women. Consequently, the US Navy’s toxicity experts have lowered short-term exposure limits and 24-h emergency exposure limits for submarines with female crew members to 0.8% (8,000 ppm) (46). In fact, most countries have set references values between 1,000 ppm to 1800 ppm in school, and working environments (47).

Although low levels of CO_2_ have long been regarded as a toxicant with no immediate lethal effects, recent studies have sparked interest in its potential health implications, particularly concerning chronic and/or intermittent long-term exposure. Such exposure has been associated with pathological conditions, including DNA alterations, nasal inflammation, and pulmonary inflammation (48). Notably, during the SARS-CoV-2 pandemic, the wearing of face masks has contributed to increased resistance and dead space volume, resulting in the re-breathing of CO_2_. Elevated blood CO_2_ levels are a key feature of the *Mask-Induced Exhaustion Syndrome* (49). In addition, rising indoor CO_2_ concentrations have been associated with symptoms characteristic of *Sick Building Syndrome*. These symptoms include headache, drowsiness, lethargy, tiredness, mental fatigue, reduction in decision-making performance, dizziness, as well as upper respiratory and mucosal symptoms, and skin irritation, such as itching, stinging, or dryness (50–52).

Nevertheless, given the current global trend of rising outdoor atmospheric CO_2_ concentrations, it is imperative to re-evaluate the effects of low-level CO_2_ exposure on human health. Indeed, it has been documented that CO_2_ can produce various hazardous health effects even at relatively low concentrations (13, 51). Long-term exposure to air polluted with CO_2_ from soil diffuse degassing sites has been linked to an increased risk of developing pulmonary restrictive diseases and exacerbating chronic obstructive pulmonary diseases, particularly among asthmatic and older adults (53). Residents of volcanic areas chronically exposed to high concentrations of CO_2_, such as Furnas in the Azores Islands, Portugal, exhibit a high incidence of chronic bronchitis and other respiratory disorders (54). Likewise, a high prevalence of non-infectious respiratory diseases has been observed among residents living in geothermal areas, such as Rotorua, New Zealand (25). Even more so, recent studies suggest that increased CO_2_ exposure may contribute to obesity, potentially playing a role in the rising trends of obesity and diabetes after chronic exposures to around 500 ppm CO_2_ (55–57).

Considering these findings, it is worth noting that while an acceptable long-term exposure range (ALTER) of ≤3,000 ppm for CO_2_ in residential indoor air had initially been established (58), Satish reported that even indoor concentrations ranging from 3,000 to 1,000 ppm may lead to deleterious health effects. These effects include mucous membrane or respiratory symptoms, decreased test performance, and neurophysiological symptoms (e.g., headache, fatigue) (51). Gall et al. define a level of concern for CO_2_ exposure, particularly regarding adverse cognitive impacts, as average personal exposure exceeding 1,000 ppm for exposures greater than 2.5 h (59). Consequently, international agencies have adopted an acceptable long-term exposure range (ALTER) of ≤1,000 ppm for CO_2_ in residential indoor air (54, 56).

Furthermore, although not directly related to the harmful effects of CO_2_, it is pertinent to note that CO_2_ naturally carries radioactive radon gas, a well-known carcinogen (60). Long-term inhalation exposure to radon has been linked to respiratory disorders and lung cancer (61, 62). Higher cancer incidence rates have been reported in volcanic areas compared to non-volcanic areas, with CO_2_ potentially acting as a carrier for radon gas (63). Populations residing in volcanic areas with chronic exposure to environmental factors resulting from volcanic activity show a higher risk of certain cancers, such as lip, oral cavity, pharynx, and breast cancers (64). Indeed, biomonitoring studies in the Azores Islands, Portugal, have revealed a higher incidence of micronucleated cells in oral epithelium (a recognized predictive biomarker of cancer risk) in individuals inhabiting volcanically active environments, suggesting an increased risk of cancer (65), thus underscoring the need for further investigation into the health effects of low levels of CO_2_ long-term exposure. Potential adverse health effects related to long-term CO_2_ exposure are summarized in Table 2.

**Table 2:** Potential chronic adverse health effects related to long-term (chronic/intermittent) CO_2_ exposure.

Mental fatigue, impaired work performance, decrements in decision-making performance
Development of restrictive lung diseases,
Exacerbation of chronic obstructive pulmonary diseases
Increased incidence of chronic bronchitis
Potential contribution to obesity and diabetes
DNA damage
Elevated cancer risk
Lung cancer

DNA, Deoxyribonucleic Acid.

## 3 Discussion

In light of these considerations, akin to urban populations residing in highly polluted regions, it is evident that safeguarding the inhabitants of volcanic areas from the adverse effects associated with relatively low-dose/long-term CO_2_ inadvertent exposure is imperative.

In volcanic regions featuring degassing sites, all the subtle/chronic effects of CO_2_ must be duly considered by Public Health Authorities when implementing measures aimed at reducing gas concentrations and mitigating the associated health risks. While ventilation with ambient air is a common strategy for reducing indoor CO_2_ levels, it may not always be suitable in volcanic degassing sites where outdoor CO_2_ levels are also elevated. Consequently, in such instances, evacuating volcanic/geothermal areas may be necessary, mirroring the evacuation ordered in the Port of Volcano (Volcano Island, Italy) in 2021 when outdoor CO_2_ levels of approximately 12.5% (125.000 ppm) were recorded (66). This proactive approach should be prioritized by Public Health Authorities in volcanic regions where sporadic and unpredictable releases of CO_2_ may occur, resulting in inadvertent population exposure to escalating CO_2_ levels (27).

Moreover, the existence of population subgroups more susceptible to the toxic effects of CO_2_ must also be taken into consideration. For instance, the exposure of older adults, pregnant women, cardiac or pulmonary patients, and children to CO_2_ warrants careful oversight (46, 49).

In this scenario, in volcanic regions it would be necessary to be able to set dangerous levels at which chronic CO_2_ exposure capable of producing subtle health effects is occurring. Unfortunately, measurement of bicarbonate levels or blood pH in populations chronically exposed to increasing CO_2_ concentrations is neither useful, nor possible, as arterial blood gasometry tests are invasive and cannot be repeated continuously for an individual tracking. Similarly, while the quantification of exhaled CO_2_ through capnography is valuable for assessing hypoventilation, there is no scientific evidence supporting its efficacy in diagnosing CO_2_ intoxication or exposure. In fact, to diagnose chronic or even acute CO_2_ intoxication, exposure to this gas must be confirmed in a manner akin to practices in forensic and occupational medicine (15, 19), namely, by quantifying the CO2 levels in the suspected area. In any case, in long-term exposures, it may be possible to rely on the presence of neurophysiological symptoms, (e.g., headache, or fatigue), and to perform decision-making tests, as described by Satish et al.

Implementing measures to protect the population from anthropogenic high indoor CO_2_ levels is feasible, given the extensive regulations and recommendations governing indoor CO_2_ exposure levels both internationally and occupationally. However, mitigating the effects or controlling emissions from outdoor CO_2_ sources poses significant challenges, being enormously complex and often hardly feasible.

Indeed, outdoor CO_2_ concentrations are not typically regulated, and research into the toxic effects of CO_2_ in open environments remains scarce. In the context of volcanic degassing sites, Public Health faces several challenges. These include the inability to control emission sources, which often produce unpredictable peaks of extremely high CO_2_ levels, and the absence of regulatory thresholds. This is due to limited evidence on the health effects of CO_2_ exposure on the general population, both short- and long-term, which complicates decision-making processes.

Further prospective studies are warranted to advance our understanding of the chronic health effects of volcanic emissions, including gases like CO_2_, as well as aerosols and ashes. Ongoing studies, such as the studies on the island of La Palma (Spain), could be instrumental in defining safe versus dangerous concentrations of CO_2_ for the population (65, 66). This would enable International Agencies and institutions to establish guidelines and recommendations for protecting health in outdoor areas affected by volcanic gas emissions. Such insights would be invaluable for Local Authorities in conducting health impact assessments and making informed planning decisions, especially concerning the placement of human settlements in volcanic zones.

In summary, although the scientific evidence on lethal incidents due to CO_2_ degassing in volcanic zones is generally limited, the potential for such incidents cannot be ignored, particularly in active volcanic areas where extraordinarily high concentrations of CO_2_ have been recorded both indoors and outdoors. This is particularly concerning in areas with significant residential and tourist activity.

Considering the “precautionary principle” in health impact assessments, which emphasizes the avoidance of unnecessary risks to population health (67). In case of population exposed to volcanic emissions, Public Health Authorities have adopted several measures to prevent or minimize individual hazards. These measures included: (a) Establishing a sensor network to continuously monitor CO_2_ concentrations indoors and outdoors (68); (b) Strictly controlling access to areas with the highest CO_2_ concentrations to mitigate the risk of accidental exposures in areas with elevated (potentially lethal) CO_2_ levels (66); (c) Prioritizing efforts to keep vulnerable populations (children, older adults, pregnant women, individuals with pre-existing respiratory or cardiovascular conditions) away from such areas (49); (d) Enhancing information dissemination to affected populations (including tourists) through informative meetings to foster cooperation and shared responsibility for the implemented measures (40). Thus, raising awareness and disseminating information about the “hidden” dangers of CO_2_ is essential.

Finally, as highlighted by Pefferkorn et al., it is crucial not to overlook the importance of adhering to safety protocols and utilizing appropriate respiratory protection near eruptive zones, even in open environments, due to the potential invisible dangers posed by volcanic areas (69).
